# Supplementary figures and images for: Response of arbuscular mycorrhizal fungal community in soil and roots to grazing differs in a wetland on the Qinghai-Tibet plateau
Source: PeerJ. 2020 Jun 19;8:e9375. doi: 10.7717/peerj.9375 (PMC7307571; doi:10.7717/peerj.9375)

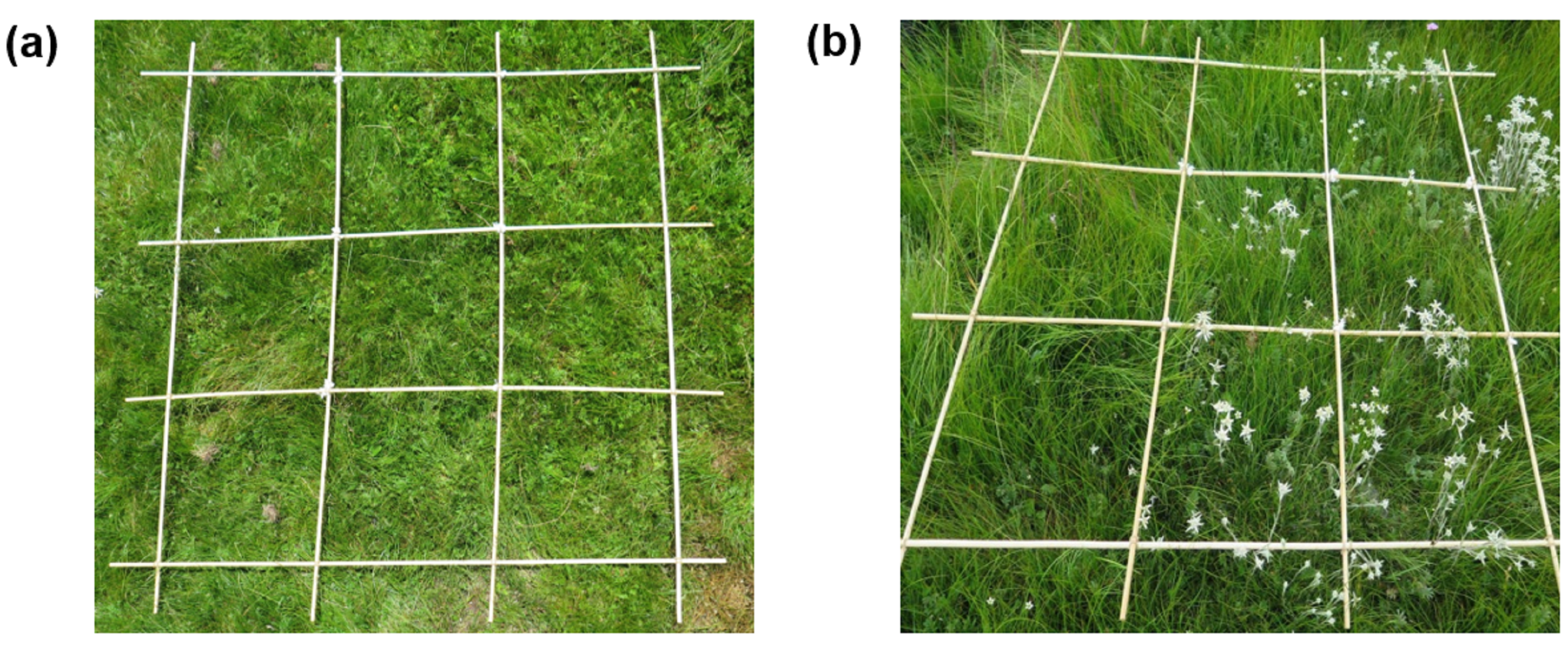

Supplement: Supplemental Information 1 — (a) vegetation in grazing plots, (b) vegetation in non-grazing plots. [file peerj-08-9375-s001.png]

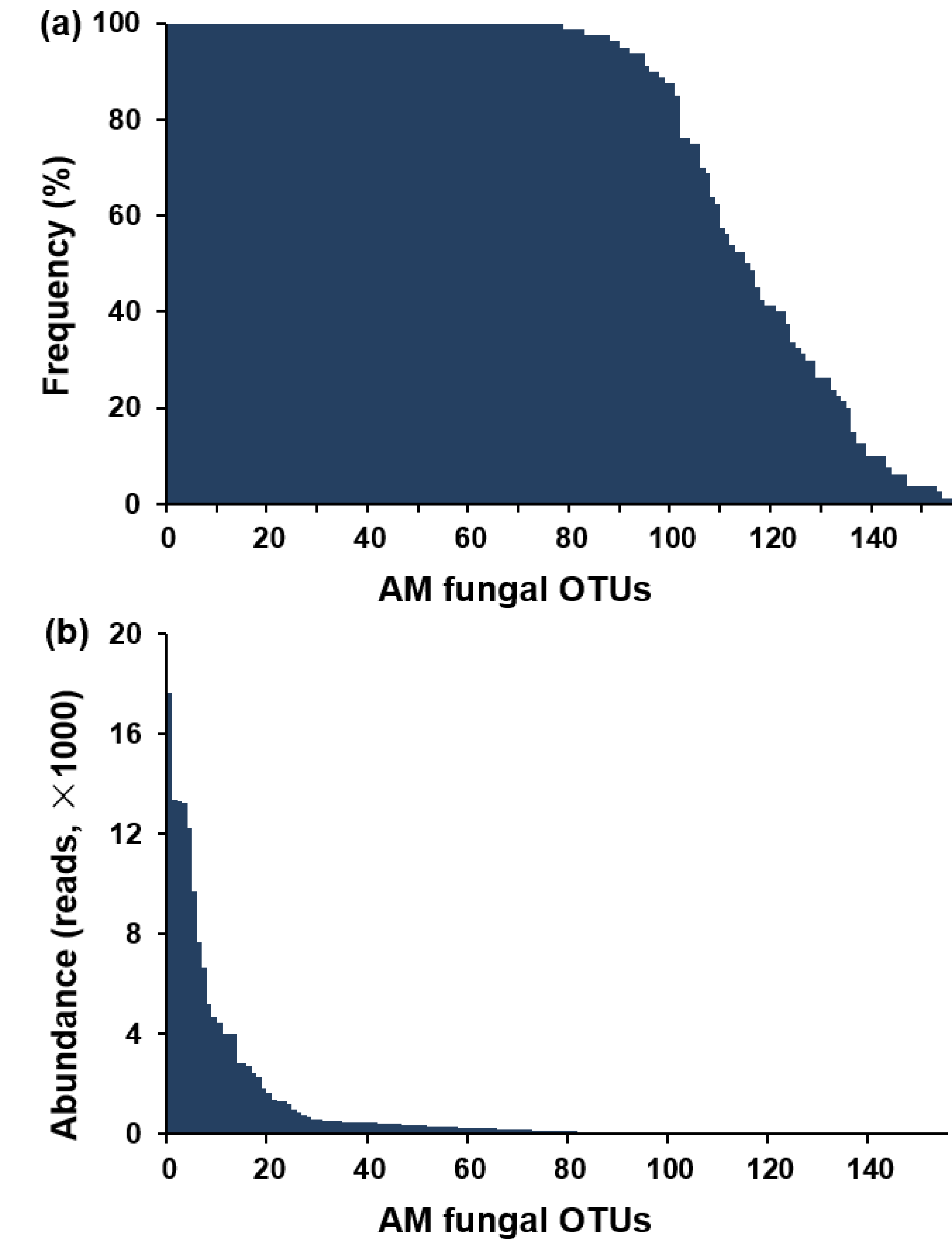

Supplement: Supplemental Information 2 [file peerj-08-9375-s002.png]

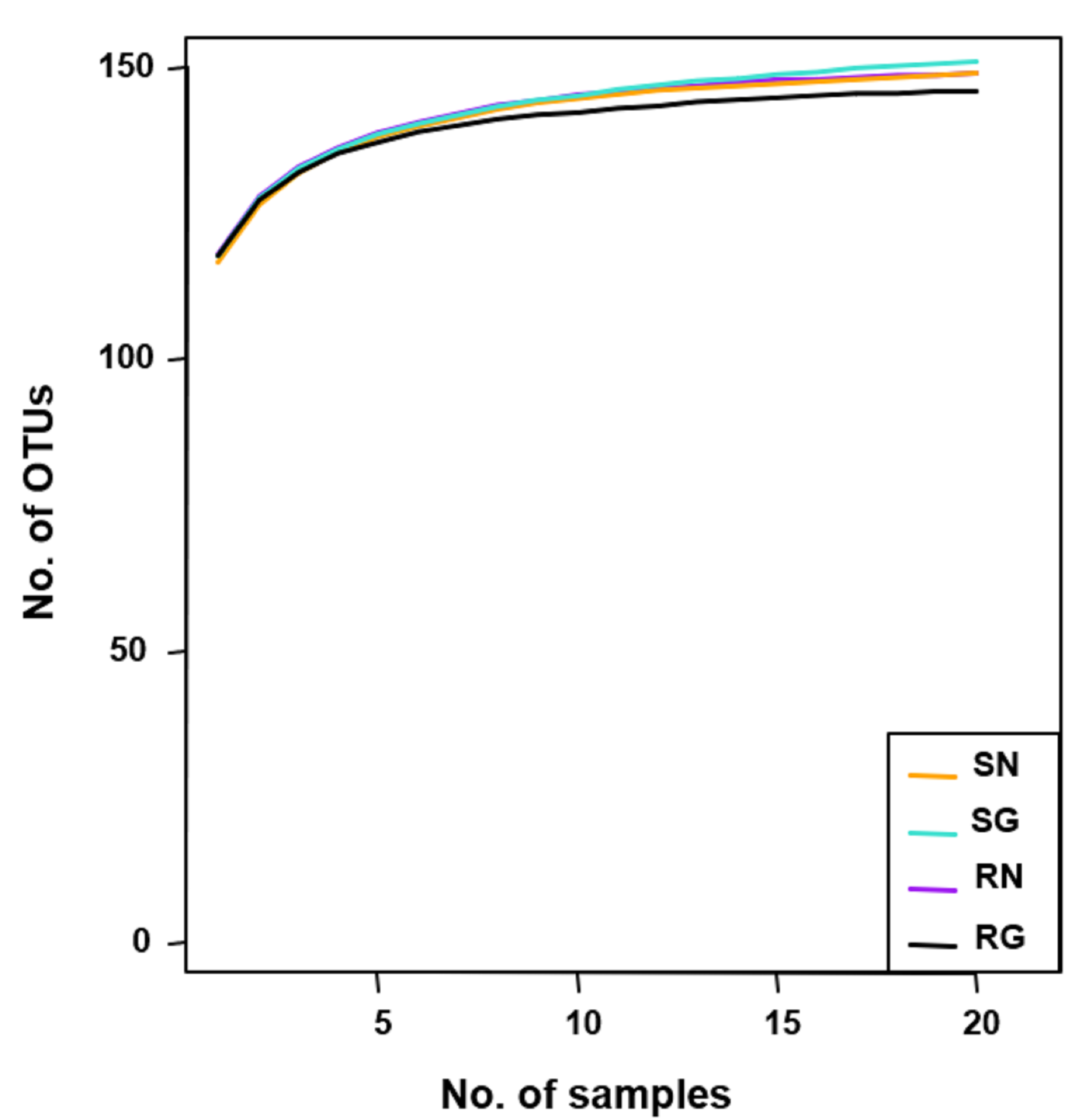

Supplement: Supplemental Information 3 — SN, soil non-grazing; SG, soil grazing; RN, root non-grazing; RG, root grazing. [file peerj-08-9375-s003.png]
