# Supplementary material for: Response of arbuscular mycorrhizal fungal community in soil and roots to grazing differs in a wetland on the Qinghai-Tibet plateau
Source: PeerJ. 2020 Jun 19;8:e9375. doi: 10.7717/peerj.9375 (PMC7307571; doi:10.7717/peerj.9375)
Supplement: Supplemental Information 5 [file peerj-08-9375-s005.docx]

**Table S2** General linear model (GLM) showing the effect of grazing and sample type (soil and root) on the operational taxonomic unit richness of arbuscular mycorrhizal fungi.

|  | Estimate | SE | t-value | *P*-value |
| --- | --- | --- | --- | --- |
| Grazing | 0.45 | 1.0258 | 0.439 | 0.662 |
| Grazing: Sample type | 6.15 | 1.0258 | 5.995 | < 0.001 |
| Non-grazing: Sample type | 4.85 | 1.0258 | 4.728 | < 0.001 |
