# Supplementary material for: Response of arbuscular mycorrhizal fungal community in soil and roots to grazing differs in a wetland on the Qinghai-Tibet plateau
Source: PeerJ. 2020 Jun 19;8:e9375. doi: 10.7717/peerj.9375 (PMC7307571; doi:10.7717/peerj.9375)
Supplement: Supplemental Information 6 — G, grazing; NG, non-grazing; ST, sample type. [file peerj-08-9375-s006.docx]

**Table S3** General linear model (GLM) showing the effect of grazing and sample type (soil and root) on the relative abundance of arbuscular mycorrhizal fungal operational taxonomic units. G, grazing; NG, non-grazing; ST, sample type.

| OTU | Variable | Estimate | SE | t-value | *P*-value |
| --- | --- | --- | --- | --- | --- |
| OTU5 | G | -9.997 | 12.376 | -0.808 | 0.422 |
|  | G: ST | -23.43 | 10.556 | -2.22 | 0.029 |
|  | NG: ST | -14.083 | 7.432 | -1.895 | 0.062 |
| OTU8 | G | -10.933 | 10.071 | -1.086 | 0.281 |
|  | G: ST | -24.08 | 8.704 | -2.767 | 0.007 |
|  | NG: ST | -13.065 | 6.362 | -2.054 | 0.043 |
| OTU23 | G | 36.07 | 42.83 | 0.842 | 0.402 |
|  | G: ST | -57.3 | 25.79 | -2.222 | 0.029 |
|  | NG: ST | -103.23 | 35.44 | -2.913 | 0.005 |
| OTU25 | G | 29.007 | 11.774 | 2.464 | 0.016 |
|  | G: ST | -5.867 | 5.925 | -0.99 | 0.325 |
|  | NG: ST | -28.189 | 11.844 | -2.38 | 0.020 |
| OTU141 | G | 43.13 | 24.94 | 1.73 | 0.088 |
|  | G: ST | -29.32 | 12.92 | -2.27 | 0.026 |
|  | NG: ST | -50.29 | 24.19 | -2.079 | 0.041 |
| OTU17 | G | -15.97 | 55.97 | -0.285 | 0.776 |
|  | G: ST | -93.62 | 43.69 | -2.143 | 0.035 |
|  | NG: ST | -75.43 | 38.96 | -1.936 | 0.057 |
| OTU18 | G | -0.4582 | 1.7129 | -0.268 | 0.790 |
|  | G: ST | 48.4262 | 9.9215 | 4.881 | < 0.001 |
|  | NG: ST | 54.1705 | 10.8433 | 4.996 | < 0.001 |
| OTU4 | G | 0.09932 | 1.48769 | 0.067 | 0.947 |
|  | G: ST | 6.83523 | 1.79088 | 3.817 | 0.000 |
|  | NG: ST | 10.02304 | 1.95419 | 5.129 | < 0.001 |
| OTU14 | G | -0.5703 | 2.2627 | -0.252 | 0.802 |
|  | G: ST | 9.8952 | 2.7378 | 3.614 | < 0.001 |
|  | NG: ST | 13.9932 | 2.892 | 4.839 | < 0.001 |
| OTU12 | G | 73.727 | 33.251 | 2.217 | 0.030 |
|  | G: ST | 79.447 | 35.057 | 2.266 | 0.026 |
|  | NG: ST | 45.489 | 56.99 | 0.798 | 0.427 |
| OTU7 | G | -1.529 | 4.813 | -0.318 | 0.752 |
|  | G: ST | 16.639 | 5.603 | 2.969 | 0.004 |
|  | NG: ST | 26.273 | 5.932 | 4.429 | < 0.001 |
