# Supplementary material for: Response of arbuscular mycorrhizal fungal community in soil and roots to grazing differs in a wetland on the Qinghai-Tibet plateau
Source: PeerJ. 2020 Jun 19;8:e9375. doi: 10.7717/peerj.9375 (PMC7307571; doi:10.7717/peerj.9375)
Supplement: Supplemental Information 7 [file peerj-08-9375-s007.docx]

**Table S4** Comparison of the relative abundance of abundant arbuscular mycorrhizal (AM) fungal operational taxonomic units (OTUs, relative abundance > 1%) in soil and roots in grazing and non-grazing treatments.

| OTU | Taxonomic position | RN | RG | SN | SG |
| --- | --- | --- | --- | --- | --- |
| OTU5 | Glomerales | 4.575 ± 2.432 bc | 3.139 ± 0.730 c | 12.866 ± 2.871 a | 11.872 ± 1.845 ab |
| OTU8 | Diversisporales | 4.018 ± 1.312 b | 2.792 ± 0.718 b | 8.459 ± 1.240 a | 8.518 ± 1.177 a |
| OTU23 | Archaeosporales | 0.831 ± 0.116 b | 1.186 ± 0.480 b | 5.834 ± 1.384 a | 3.705 ± 1.172 a |
| OTU25 | Glomerales | 1.926 ± 0.391 b | 4.364 ± 1.348 a | 4.214 ± 0.678 a | 5.866 ± 0.576 a |
| OTU141 | Glomerales | 1.062 ± 0.303 b | 1.958 ± 0.473 b | 2.277 ± 0.507 a | 4.599 ± 0.763 a |
| OTU17 | Diversisporales | 0.877 ± 0.241 b | 0.770 ± 0.221 b | 2.594 ± 0.994 a | 2.753 ± 0.931 a |
| OTU18 | Glomerales | 15.164 ± 3.600 a | 14.179 ± 3.178 a | 1.646 ± 0.183 b | 1.802 ± 0.150 b |
| OTU4 | Glomerales | 5.582 ± 0.408 a | 5.613 ± 0.370 a | 3.579 ± 0.205 b | 4.056 ± 0.120 b |
| OTU14 | Glomerales | 3.782 ± 0.282 a | 3.702 ± 0.259 a | 2.473 ± 0.137 b | 2.710 ± 0.077 b |
| OTU12 | Glomerales | 1.010 ± 0.128 b | 3.950 ± 2.278 a | 0.692 ± 0.134 b | 0.955 ± 0.183 b |
| OTU7 | Glomerales | 1.735 ± 0.121 a | 1.690 ± 0.102 a | 1.192 ± 0.076 b | 1.319 ± 0.043 b |

Data (means ± SE) in the same row with different letters are significantly different at *P* < 0.05, as indicated by Conover’s test. SN, soil non-grazing; SG, soil grazing; RN, root non-grazing; RG, root grazing.
