# Supplementary material for: Response of arbuscular mycorrhizal fungal community in soil and roots to grazing differs in a wetland on the Qinghai-Tibet plateau
Source: PeerJ. 2020 Jun 19;8:e9375. doi: 10.7717/peerj.9375 (PMC7307571; doi:10.7717/peerj.9375)
Supplement: Supplemental Information 8 — G, grazing; NG, non-grazing; ST, sample type. [file peerj-08-9375-s008.docx]

**Table S5** General linear model (GLM) showing the effect of grazing and sample type (soil and root) on the relative abundance of arbuscular mycorrhizal fungal orders. G, grazing; NG, non-grazing; ST, sample type.

| Order | Variable | Estimate | SE | t-value | *P*-value |
| --- | --- | --- | --- | --- | --- |
| Glomerales | G | 0.07158 | 0.05356 | 1.337 | 0.185 |
|  | G: ST | 0.19417 | 0.05654 | 3.434 | < 0.001 |
|  | NG: ST | 0.24641 | 0.06114 | 4.03 | < 0.001 |
| Diversisporales | G | -7.472 | 3.659 | -2.042 | 0.045 |
|  | G: ST | -10.498 | 3.422 | -3.068 | 0.003 |
|  | NG: ST | -3.504 | 2.207 | -1.588 | 0.116 |
| Archaeosporales | G | 1.539 | 7.59 | 0.203 | 0.840 |
|  | G: ST | -8.987 | 5.814 | -1.546 | 0.126 |
|  | NG: ST | -13.547 | 5.878 | -2.305 | 0.024 |
| Paraglomerales | G | -2357.98 | 1255.63 | -1.878 | 0.064 |
|  | G: ST | -2123.19 | 1264.42 | -1.679 | 0.097 |
|  | NG: ST | 12.25 | 46.74 | 0.262 | 0.794 |
