# Supplementary material for: Response of arbuscular mycorrhizal fungal community in soil and roots to grazing differs in a wetland on the Qinghai-Tibet plateau
Source: PeerJ. 2020 Jun 19;8:e9375. doi: 10.7717/peerj.9375 (PMC7307571; doi:10.7717/peerj.9375)
Supplement: Supplemental Information 9 [file peerj-08-9375-s009.docx]

**Table S6** Nested permutational multivariate analysis of variance (PerMANOVA) testing the effect of grazing and sample type (root and soil) on arbuscular mycorrhizal fungal community composition.

|  | Df | SS | MS | *F* | *R*^2^ | *P*-value |
| --- | --- | --- | --- | --- | --- | --- |
| Grazing | 1 | 0.07179 | 0.071794 | 2.3394 | 0.02518 | 0.012 |
| Grazing:Sample type | 2 | 0.44705 | 0.223525 | 7.2836 | 0.15679 | 0.001 |
| Residuals | 76 | 2.33234 | 0.030689 |  | 0.81803 |  |
| Total | 79 | 2.85119 |  |  | 1 |  |
