# Supplementary material for: Response of arbuscular mycorrhizal fungal community in soil and roots to grazing differs in a wetland on the Qinghai-Tibet plateau
Source: PeerJ. 2020 Jun 19;8:e9375. doi: 10.7717/peerj.9375 (PMC7307571; doi:10.7717/peerj.9375)
Supplement: Supplemental Information 10 [file peerj-08-9375-s010.docx]

**Table S7** Permutational multivariate analysis of variance (PerMANOVA) testing the effect of grazing on arbuscular mycorrhizal fungal community composition in soil.

|  | Df | SS | MS | *F* | *R*^2^ | *P*-value |
| --- | --- | --- | --- | --- | --- | --- |
| Grazing | 1 | 0.01947 | 0.01947 | 1.985 | 0.04964 | 0.006 |
| Residuals | 38 | 0.37278 | 0.00981 |  | 0.95036 |  |
| Total | 39 | 0.39225 |  |  | 1 |  |
