# Supplementary material for: Response of arbuscular mycorrhizal fungal community in soil and roots to grazing differs in a wetland on the Qinghai-Tibet plateau
Source: PeerJ. 2020 Jun 19;8:e9375. doi: 10.7717/peerj.9375 (PMC7307571; doi:10.7717/peerj.9375)
Supplement: Supplemental Information 11 [file peerj-08-9375-s011.docx]

**Table S8** Permutational multivariate analysis of variance (PerMANOVA) testing the effect of grazing on arbuscular mycorrhizal fungal community composition in roots.

|  | Df | SS | MS | *F* | *R*^2^ | *P*-value |
| --- | --- | --- | --- | --- | --- | --- |
| Grazing | 1 | 0.01155 | 0.01155 | 1.094 | 0.028 | 0.335 |
| Residuals | 38 | 0.40133 | 0.01056 |  | 0.972 |  |
| Total | 39 | 0.41288 |  |  | 1 |  |
